# Unique Role of the WD-40 Repeat Protein 5 (WDR5) Subunit within the Mixed Lineage Leukemia 3 (MLL3) Histone Methyltransferase Complex*

**DOI:** 10.1074/jbc.M115.684142

**Published:** 2015-08-31

**Authors:** Stephen A. Shinsky, Michael S. Cosgrove

**Affiliations:** From the Department of Biochemistry and Molecular Biology, State University of New York Upstate Medical University, Syracuse, New York 13210

**Keywords:** chromatin, enzyme, histone, histone methylation, leukemia

## Abstract

The MLL3 (mixed lineage leukemia 3) protein is a member of the human SET1 family of histone H3 lysine 4 methyltransferases and contains the conserved WDR5 interaction (Win) motif and the catalytic suppressor of variegation, enhancer of zeste, trithorax (SET) domain. The human SET1 family includes MLL1–4 and SETd1A/B, which all interact with a conserved subcomplex containing WDR5, RbBP5, Ash2L, and DPY-30 (WRAD) to form the minimal core complex required for full methyltransferase activity. However, recent evidence suggests that the WDR5 subunit may not be utilized in an identical manner within all SET1 family core complexes. Although the roles of WDR5 within the MLL1 core complex have been extensively studied, not much is known about the roles of WDR5 in other SET1 family core complexes. In this investigation, we set out to characterize the roles of the WDR5 subunit in the MLL3 core complex. We found that unlike MLL1, the MLL3 SET domain assembles with the RbBP5/Ash2L heterodimer independently of the Win motif-WDR5 interaction. Furthermore, we observed that WDR5 inhibits the monomethylation activity of the MLL3 core complex, which is dependent on the Win motif. We also found evidence suggesting that the WRAD subcomplex catalyzes weak H3K4 monomethylation within the context of the MLL3 core complex. Furthermore, solution structures of the MLL3 core complex assembled with and without WDR5 by small angle x-ray scattering show similar overall topologies. Together, this work demonstrates a unique role for WDR5 in modulating the enzymatic activity of the MLL3 core complex.

## Teaser

**Background:** The mixed lineage leukemia 3 (MLL3) core complex catalyzes monomethylation of histone H3 lysine 4 (H3K4).

**Results:** The WDR5 subunit is not required for MLL3 core complex assembly and partially inhibits methyltransferase activity.

**Conclusion:** WDR5 plays a unique role within the MLL3 core complex.

**Significance:** SET1 family complexes retain WDR5 for distinct purposes.

## Introduction

Methylation at lysine 4 of histone H3 (H3K4) is a highly conserved post-translational modification involved in the epigenetic regulation of eukaryotic transcription (1). Misregulation of H3K4 methylation is associated with different cancers and developmental disorders; however, the mechanisms involved are not fully understood (2). In humans, enzymes that catalyze H3K4 methylation include the SET1 family of histone methyltransferases, which consists of the mixed lineage leukemia (MLL) proteins 1–4 and SETd1A/B (3–10). The SET1 family members contain the evolutionarily conserved suppressor of variegation, enhancer of zeste, trithorax (SET)^2^ domain, which catalyzes monomethylation of H3K4 (11–15). Full enzymatic activity requires interaction of the SET domain with the conserved WRAD subcomplex containing WD-40 repeat protein-5 (WDR5), retinoblastoma-binding protein-5 (RbBP5), absent small homeotic-2-like (Ash2L), and dumpy-30 (DPY-30) (16–19). A SET1 family member assembled with WRAD is known as the core complex, which represents the minimal complex required for full H3K4 methyltransferase activity *in vitro* and *in vivo* (16, 20). A central question is to understand how WRAD controls the activity of SET1 family proteins and contributes to the regulation of H3K4 methylation in cells.

MLL1 is the most studied human SET1 family member and has served as a model for how other SET1 family core complexes function. Biochemical reconstitution experiments show that the isolated MLL1 SET domain is a slow H3K4 monomethyltransferase (20). In contrast, the complex containing MLL1 and WRAD is a relatively fast H3K4 mono- and dimethyltransferase (20). The dimethylation activity of the MLL1 core complex depends on the WDR5 subunit of WRAD, which binds with high affinity to a conserved arginine residue within the WDR5 interaction (Win) motif of MLL1 located in a region N-terminal to the SET domain (20, 21). The WDR5-MLL1 interaction is critical for the assembly of the MLL1 core complex, as the RbBP5 and Ash2L subunits do not stably interact with MLL1 in the absence of WDR5 (20, 21). Furthermore, mutation of the conserved Win motif arginine to alanine or leucine prevents complex assembly and abolishes the H3K4 dimethylation activity of the MLL1 core complex (21, 22). Because the Win motif is conserved in all metazoan SET1 family members (21, 23), these results suggest that WDR5 functions to bridge the interaction between the SET1 family SET domain and the RbBP5-Ash2L heterodimer within each core complex, which is critical for formation of the H3K4 dimethylation active site.

However, although this paradigm holds true for most SET1 family complexes, recent experiments uncovered differences among family members suggesting each has evolved unique mechanisms of action and modes of regulation. Biochemical reconstitution experiments reveal that although the MLL1 and MLL4 complexes catalyze predominantly H3K4 mono- and dimethylation, the SETd1A and SETd1B complexes catalyze mono-, di-, and trimethylation (24). In contrast, whereas the MLL2 and MLL3 complexes have stimulated monomethyltransferase activity in the presence of WRAD, they do not catalyze appreciable amounts of H3K4 di- and trimethylation (24). In addition, although interaction with the WRAD subcomplex stimulates the enzymatic activity of all human SET1 family core complexes, SET1 family members differ in their requirement for WDR5 (24). For instance, loss of WDR5 results in decreased H3K4 dimethyltransferase activity catalyzed by the MLL1 core complex, but it increases H3K4 monomethylation activity by the MLL3 core complex (24). MLL3 is the only human SET1 family member whose activity is stimulated by loss of WDR5 from the core complex (24). These results suggest that MLL3 interacts with WRAD in a manner that is distinct from that of the other SET1 family members.

To better understand this paradox, in this investigation we further characterized the complex assembly, enzymatic activity, and solution structure of the MLL3 core complex in the presence and absence of WDR5. We found that unlike MLL1, the MLL3 SET domain stably interacts with the RbBP5/Ash2L heterodimer in the absence of WDR5. We found that the MLL3 core complex lacking WDR5 displays ∼100-fold more H3K4 monomethylation activity compared with that of the complex assembled with stoichiometric amounts of WDR5. We also present evidence that WRAD catalyzes monomethylation within the MLL3 core complex, an activity that is stimulated in the absence of WDR5. Small angle x-ray scattering (SAXS) and analytical ultracentrifugation (AUC) experiments indicate that MLL3 complexes assembled with and without WDR5 have relatively similar structural features in solution and overall stabilities. However, we found that the MLL3 Win motif is conserved, interacts with WDR5 with high affinity *in vitro*, and contributes to stabilizing the interaction of WDR5 within the core complex. These results suggest that similar to all SET1 family complexes, WDR5 has been retained within the MLL3 core complex for some vital function, such as gene targeting. However, unlike that of other SET1 family complexes, WDR5 is less important for complex stability and plays a unique role in inhibiting the enzymatic activity of the MLL3 core complex. These results suggest that modulation of the WDR5-MLL3 interaction may be a unique mode of regulation of the enzymatic activity of the MLL3 core complex.

## Experimental Procedures

### 

#### 

##### Peptides

A histone H3 peptide was synthesized by GeneScript and contained residues 1–20 followed by GGK-biotin. The peptide was blocked by amidation of the C terminus and purified to 95% purity.

##### Protein Expression/Purification

A human MLL1 (UniProtKB ID Q03164) construct consisting of residues 3745–3969 and a human MLL3 (UniProtoKB ID Q8NEZ5) construct consisting of residues 4690–4911 were subcloned into pGST parallel expression vectors (25), individually expressed in *Escherichia coli* (Rosetta 2 (DE3) pLysS; Novagen) using 1 mm isopropyl 1-thio-β-d-galactopyranoside, and grown at 16 °C for 26 h. Proteins were purified over a GSTrap-FF column (GE Healthcare) and eluted with a gradient of 0–10 mm reduced glutathione. Pooled fractions were treated with GST-tagged tobacco etch virus protease and dialyzed at 4 °C with three changes into a buffer containing 50 mm Tris (pH 7.3), 300 mm NaCl, 10% glycerol, 3 mm dithiothreitol (DTT), and 1 μm ZnCl_2_. Dialyzed protein was then passed through a GSTrap-FF column to remove uncleaved protein and tobacco etch virus protease. As a final step of purification, proteins were passed through a HiLoad 16/60 S200 size exclusion column (GE Healthcare) that was pre-equilibrated with a buffer containing 20 mm Tris (pH 7.5), 300 mm NaCl, 1 mm tris(2-carboxyethyl)phosphine, and 1 μm ZnCl_2_. Full-length WRAD subunits in pHis parallel expression vectors (25) were individually expressed in *E. coli* (Rosetta 2 (DE3) pLysS; Novagen) and purified as described previously (21, 26).The final buffer for WRAD components consisted of 20 mm Tris (pH 7.5), 300 mm NaCl, 1 mm tris(2-carboxyethyl)phosphine, and 1 μm ZnCl_2_. Aliquots of all purified proteins were stored at −80 °C. Mutant MLL3 variants were prepared by subjecting DNA constructs to site-directed mutagenesis (QuikChange II XL, Agilent), which were sequenced to ensure the presence of the intended mutation and absence of unintended mutations. Mutant proteins were expressed and purified as described above.

##### Histone Methyltransferase Assays

MALDI-TOF methyltransferase assays were performed by incubating either the isolated MLL1 or MLL3 SET domains (14 μm) or stoichiometric SET domain complexes (7 μm) with 250 μm *S*-adenosylmethionine (Cayman Chemicals) and 10 μm H3 peptide at 15 °C for up to 24 h. At various time points, aliquots of the reaction were quenched with 0.5% trifluoroacetic acid. Samples were diluted 1:5 in α-cyano-4-hydroxycinnamic acid and shot on a Bruker Autoflex III mass spectrometer (State University of New York College of Environmental Science and Forestry at Syracuse) in reflectron mode. Final shots were averaged from 200 shots/position at five different positions. Relative methylation levels were quantitated using mMass (27). Reaction progress curves were globally fitted to an irreversible consecutive reactions model (Equations 1–3) in the program Dynafit (28) as described previously (20, 22). Inhibition curves were fit to a four-parameter Hill equation to determine IC_50_ values as described previously (23).

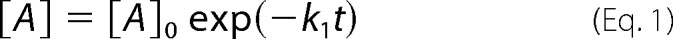


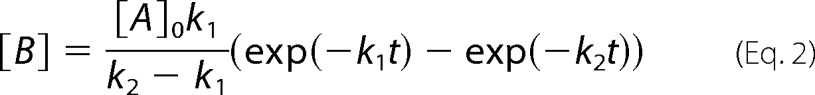


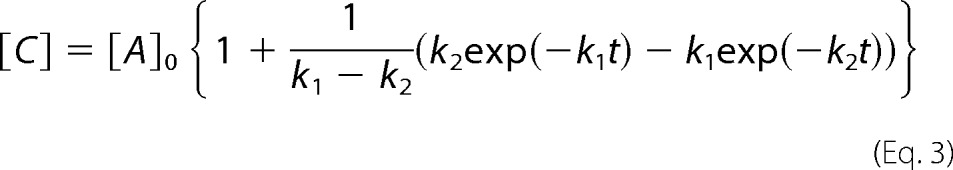


Radiometric histone methyltransferase assays were performed by incubating the isolated MLL3 SET domain in the presence or absence of WRAD/RAD (5 μm), 1.5 μCi of [^3^H]AdoMet (PerkinElmer Life Sciences, specific activity 78 Ci/mmol), and 100 μm H3 peptide for 6 h at 15 °C. All reactions were quenched with SDS loading buffer and separated by SDS-PAGE using a 4–12% BisTris gel (Invitrogen) run at 200 V for 30 min. The gels were enhanced for 30 min (Enlightning, PerkinElmer Life Sciences) and then dried for 2.5 h at 72 °C under constant vacuum. The dried gels were exposed to film (Kodak Biomax MS Film) for 4–96 h at −80 °C.

##### AUC

Isolated MLL3 proteins or MLL3 complexes were loaded into 3- or 12-mm two-sector charcoal-filled Epon centerpieces with quartz windows. All experiments were carried out using a Beckman Coulter ProteomLab XL-A analytical ultracentrifuge equipped with absorbance optics and a 4-hole An-60 Ti rotor at 60,000 rpm that was pre-equilibrated to 10 °C before each run. The samples were scanned with the time interval between scans set to zero for 100–250 scans and analyzed by the continuous distribution (*c*(*s*)) method in the program SEDFIT (29). All protein complexes were mixed in stoichiometric amounts and passed through a HiLoad 16/60 S200 size exclusion column (GE Healthcare) prior to AUC analysis. The program SEDNTERP was used to convert all experimental sedimentation coefficients to standard conditions at 20 °C in water (*s*_20,_*_w_*) (30). SEDNTERP was also used to calculate the frictional ratios (*f*/*f*_0_) using the expected molecular weight and partial specific volume based on the primary amino acid sequence (30).

##### [^3^H]AdoMet Cross-linking Assay

Purified MLL3 SET domains (wild type and mutant) alone (5 μm) or assembled with a stoichiometric amount of WRAD/RAD (5 μm) were incubated with 1.5 μCi of [^3^H]AdoMet (PerkinElmer Life Sciences, specific activity 78 Ci/mmol) at 15 °C for 3 h and then incubated on ice for 1 h. Half the volume of each sample was quenched with SDS loading buffer, and the other half was exposed to UV light (254 nm) in a Stratalinker oven at a distance of ∼15 cm for 30 min on ice. The UV-exposed samples were quenched with SDS loading buffer, and all samples were separated by SDS-PAGE using a 4–12% BisTris gel (Invitrogen) run at 200 V for 30 min. The gels were soaked in enhancer solution for 30 min (Enlightning, PerkinElmer Life Sciences) and then dried for 2.5 h at 72 °C under constant vacuum. The dried gels were exposed to film (Kodak Biomax MS Film) for 72 h at −80 °C.

##### Biological SAXS

Samples were prepared by passing individual proteins or mixed complexes over a HiLoad 16/60 S200 size exclusion column (GE HealthCare) pre-equilibrated with a buffer that contained 25 mm Tris (pH 7.5), 300 mm NaCl, 1 μm ZnCl_2_, 5% glycerol, and 3 mm dithiothreitol (DTT). Small angle x-ray scattering data were collected at Cornell High Energy Synchrotron Source G1 beam line (Ithaca, NY) using a 250-μm square x-ray beam with a flux of ∼3 × 10^11^ photons/s/mm^2^ at 9.96 keV. Measurements were made at a wavelength of 1.244 Å and at 4 °C using a dual Pilatus 100K-S detector. Data were acquired by taking 10 successive 1–2-s exposures (10–20 s total) with oscillation using 30 μl of sample using two different protein concentrations. Initial processing, including frame averaging and buffer subtraction, was done using the RAW software (31). A Guinier approximation was applied to low a *q* scattering region, and the radius of gyration (*R_g_*) was determined from a linear fit to the Guinier plot (ln(*I*) *versus q*^2^) for the *q* range that satisfies the relationship *qR_g_* <1.3 in the program Primus (ATSAS Package, EMBL) (32, 33). The pair distance distribution function (*P*(*r*)) was calculated using the indirect Fourier transform method in the program GNOM (ATSAS Package, EMBL) (34). The maximum protein dimension (*D*_max_) values were determined from the *P*(*r*) analysis, where *P*(*r*) approaches zero. We also reported the *R_g_* from the *P*(*r*) analysis. Low resolution *ab initio* envelopes were calculated from the GNOM program outputs with a high resolution limit such that *q*_max_ ≤8/*R_g_* using the program DAMMIF (ATSAS Package, EMBL) (35). Ten individual models were calculated. The program DAMAVER (ATSAS Package, EMBL) was used to align the 10 models and reject outliers (36). Outliers were defined as those models with a normalized spatial discrepancy (NSD) value greater than the average NSD ± 1 standard deviation. At most only a single model was excluded. The aligned models were further refined using the program DAMMIN (ATSAS Package, EMBL) (37). Envelope validation was done using the program HydroPro (version 10) to calculate the theoretical sedimentation coefficients of each refined average *ab initio* model and compared with experimentally determined sedimentation coefficients (38). The atomic level shell model with an atomic elements radius value of 4.4 Å was used to calculate sedimentation coefficients (38). The buffer density used was 1.0126 g/ml, and the buffer viscosity used was 0.0135 m^2^/s at 10 °C. The partial specific volume used was calculated based on amino acid sequence. The MLL3 homology model was created using Modeler with the MLL1 crystal structure as a template (PDB code 2W5Z) (39, 40). The FoxS server was used to predict the solution scattering profile of the MLL3 homology model (41). All structural figures were created using the program Chimera (42).

## Results

### 

#### 

##### WDR5 Stimulates the Activity of the MLL1 Core Complex but Inhibits the Activity of the MLL3 Core Complex

**FIGURE 1.**
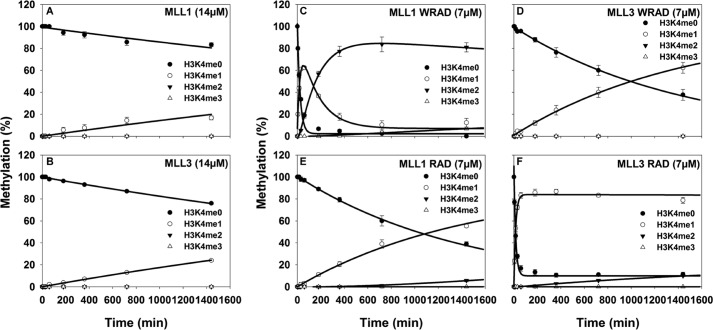
**Single turnover kinetic profiles of MLL1 and MLL3 core complexes.** *A* and *B,* reaction progress curves fitted to exponential rise/decay (see Equation 1 described under “Experimental Procedures”) from isolated MLL1 (*A*) or MLL3 (*B*) SET domains assayed at 14 μm. *C–F,* reaction progress curves for MLL1 (*C* and *E*) or MLL3 (*D* and *F*) complexes assayed at 7 μm. Progress curves were globally fitted to irreversible consecutive reaction models (Equations 1–3 in the methods) using DynaFit (28). For all plots, each time point represents the mean percentage of total integrated area for each species in MALDI-TOF reactions. *Error bars* represent ± S.E. from triplicate measurements.

We previously demonstrated that WDR5 is required to stimulate the mono- and dimethylation activities of the MLL1 core complex (20, 21). To characterize the role of WDR5 in the MLL3 core complex, we compared rates of methylation of MLL1 and MLL3 SET domains in the presence and absence of WRAD or RAD using quantitative MALDI-TOF mass spectrometry. When the isolated MLL1 or MLL3 SET domains (14 μm) were incubated with AdoMet and H3 peptide, both enzymes showed relatively weak H3K4 monomethylation (H3K4me1) activity with similar pseudo first-order rate constants (Fig. 1, *A* and *B*). These results are consistent with our previous finding that the isolated MLL1 and MLL3 SET domains predominantly catalyze H3K4 monomethylation (20, 24).

In contrast, when gel filtration-purified MLL1 and MLL3 core complexes (7 μm) were compared, significant differences were observed. As demonstrated previously, the MLL1 core complex showed stimulated monomethylation and di-methylation activities in the presence of WDR5, with the accumulation of an H3K4me1 intermediate during the course of the reaction (Fig. 1*C*). These results suggest that the MLL1 core complex uses a non-processive mechanism to catalyze mono- and dimethylation of H3K4. In contrast, the MLL3 core complex in the presence of WDR5 shows only H3K4 monomethylation activity that is stimulated in comparison with the isolated MLL3 SET domain but is ∼64-fold slower than the monomethylation rate catalyzed by the MLL1 core complex (Fig. 1*D* and Table 1). As noted previously, the MLL3 core complex does not catalyze H3K4 di- or trimethylation under the conditions assayed (24).

**TABLE 1:** **Summary of single turnover rate constants**

Complex	Rate constants*^a^*	
	*k*_1_	*k*_2_
	*h*^−*1*^	
MLL1 WRAD	2.62 ± 0.16	0.38 ± 0.036
MLL1 RAD	0.041 ± 0.00	NA*^b^*
MLL3 WRAD	0.041 ± 0.001	NA
MLL3 RAD	3.96 ± 0.22	NA

*^a^* Single turnover rates were obtained by global fitting to Equations 1–3 described under “Experimental Procedures.”

*^b^* NA indicates activities were either not observed or were too low to obtain a reliable rate constant.

When the assays were conducted with complexes that omitted the WDR5 subunit, the MLL1 core complex displayed a 64-fold decrease in the rate of H3K4 mono-methylation compared with the holo-MLL1 core complex and nearly abolished detectable levels of H3K4 di-methylation (Fig. 1*E* and Table 1). In contrast, the MLL3 core complex assembled without WDR5 showed a stimulated rate of H3K4 mono-methylation that was 96-fold greater than the MLL3 core complex assembled with stoichiometric amounts of WDR5 (Fig. 1*F* and Table 1). We also observed low levels of H3K4 di-methylation by the MLL3-RAD complex at later time points (Fig. 1*F*). Together, these results suggest that although WDR5 is essential for the full activity of the MLL1 core complex, it acts as an inhibitor of the enzymatic activity of the MLL3 core complex.

##### MLL3, RbBP5, and Ash2L Are the Minimal Components Required for the Full Activity of the MLL3 Core Complex

**FIGURE 2.**
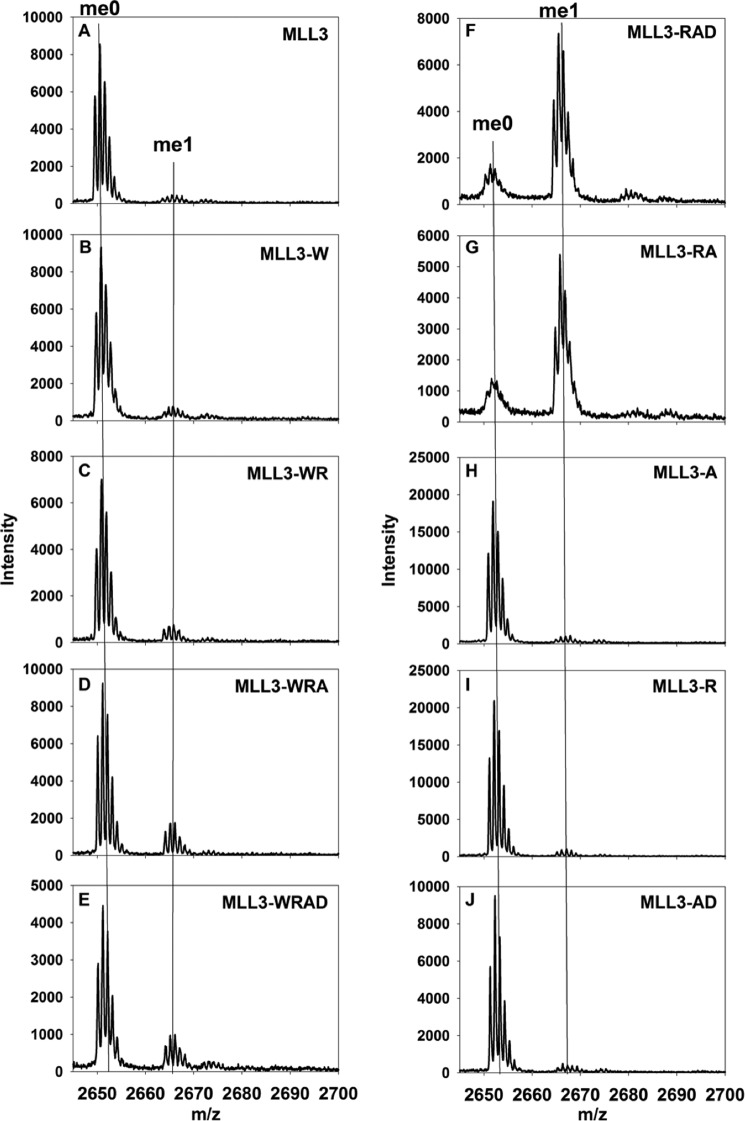
**RbBP5/Ash2L heterodimer is required for stimulation of MLL3 methyltransferase activity.** MALDI-TOF mass spectrometry showing the histone methylation of an unmodified H3 peptide after 6 h for various subcomplexes containing MLL3. *A* shows activity with the isolated MLL3 SET domain. *B–E* show complexes containing WDR5 and *F–J* show complexes lacking WDR5. The positions for unmodified (*me0*) and mono-methylated H3 peptide (*me1*) are indicated above the *graphs*.

The results from single turnover enzymatic assays demonstrated that the MLL3 core complex assembled without WDR5 is more active than the complex assembled with WDR5. To determine the minimal components necessary to stimulate the methyltransferase activity of MLL3, we assayed various MLL3 subcomplexes using quantitative MALDI-TOF mass spectrometry to compare amounts of H3K4 monomethylation after 6 h. We observed that addition of WDR5 or addition of the WDR5/RbBP5 heterodimer to MLL3 only slightly stimulated the activity of MLL3 SET domain compared with the activity of the isolated MLL3 SET domain (Fig. 2, *A–C*). However, we observed a modest stimulation in methyltransferase activity when MLL3 was assayed in the presence of the WDR5-RbBP5-Ash2L subcomplex (MLL3-WRA) (Fig. 2*D*). Addition of DPY-30 to the MLL3-WRA complex did not further stimulate methyltransferase activity (Fig. 2*E*).

Strikingly, in the absence of WDR5, the MLL3-RAD complex converted most of the peptide into the mono-methyl form after 6 h (Fig. 2*F*). A similar result was obtained with the MLL3-RA complex (Fig. 2*G*). Although the addition of the RbBP5/Ash2L heterodimer to MLL3 was sufficient for full activity, neither individual subunit was capable of stimulating activity when added to the MLL3 SET domain alone (Fig. 2, *H* and *I*). Similarly, addition of the Ash2L-DPY-30 complex to MLL3 was not sufficient for stimulation of activity (Fig. 2*J*). Together, these results suggest that the RbBP5/Ash2L heterodimer interacts with the MLL3 SET domain in the absence of WDR5 and stimulates the methyltransferase activity of the MLL3 core complex *in vitro*.

##### MLL3 Interacts with the RbBP5/Ash2L Heterodimer in the Absence of WDR5

**FIGURE 3.**
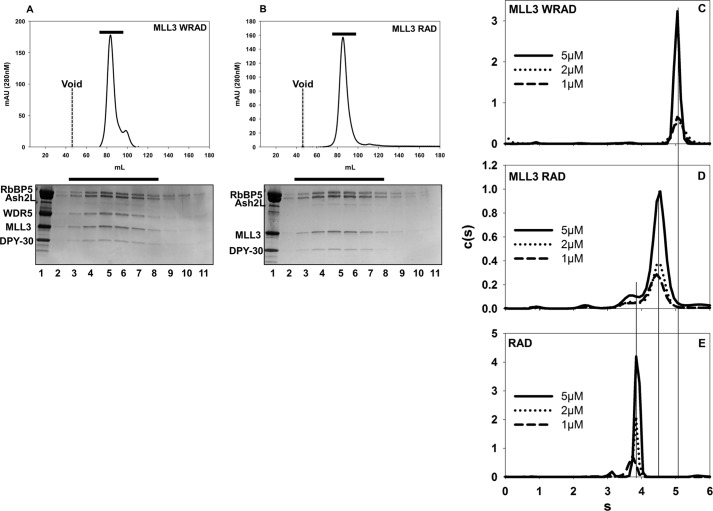
**MLL3 assembles core complexes with and without WDR5.** *Upper panels* show the size exclusion chromatographs of MLL3-WRAD (*A*) or of MLL3-RAD (*B*) passed over a Superdex 200 column (GE Healthcare). The void volume (45 mls) is indicated on the *graphs* by a *dashed line,* and the *black bars* represent the part of the elution profiles analyzed by SDS-PAGE. The *lower panels* show Coomassie Blue-stained SDS-PAGE analysis of fractions collected from under the peaks (*black bars*). *Lane 1* of each gel shows the input sample. Diffusion-free sedimentation coefficient distributions (*c*(*s*)) were derived from SV-AUC experiments of the MLL3-WRAD (*C*), MLL3-RAD (*D*), or RAD (*E*) complexes at various concentrations.

The results presented above suggest that, unlike the MLL1 SET domain, the MLL3 SET domain interacts with the RbBP5/Ash2L heterodimer in the absence of WDR5. To test this hypothesis, we compared MLL3 core complex assembly in the presence and absence of WDR5 using size exclusion chromatography and sedimentation velocity analytical ultracentrifugation (SV-AUC). When MLL3 was mixed with a stoichiometric amount of WRAD subunits and separated by size exclusion chromatography, the complex eluted mostly as a single peak with an elution volume of ∼85 ml (Fig. 3*A, upper panel*). SDS-PAGE of peak fractions showed co-elution of MLL3 with stoichiometric amounts of WRAD subunits (Fig. 3*A, lower panel*). Interestingly, when MLL3 was mixed with RAD components and passed through the size exclusion column, a single peak at the same elution volume (∼85 ml) was observed (Fig. 3*B, upper panel*). Fractions under this peak contained stoichiometric amounts of MLL3-RAD (Fig. 3*B, lower panel*). These results indicate that the MLL3-WRAD complex and the MLL3-RAD complex have similar Stokes radii and suggest that MLL3 interacts with RAD in the absence of WDR5.

**FIGURE 4.**
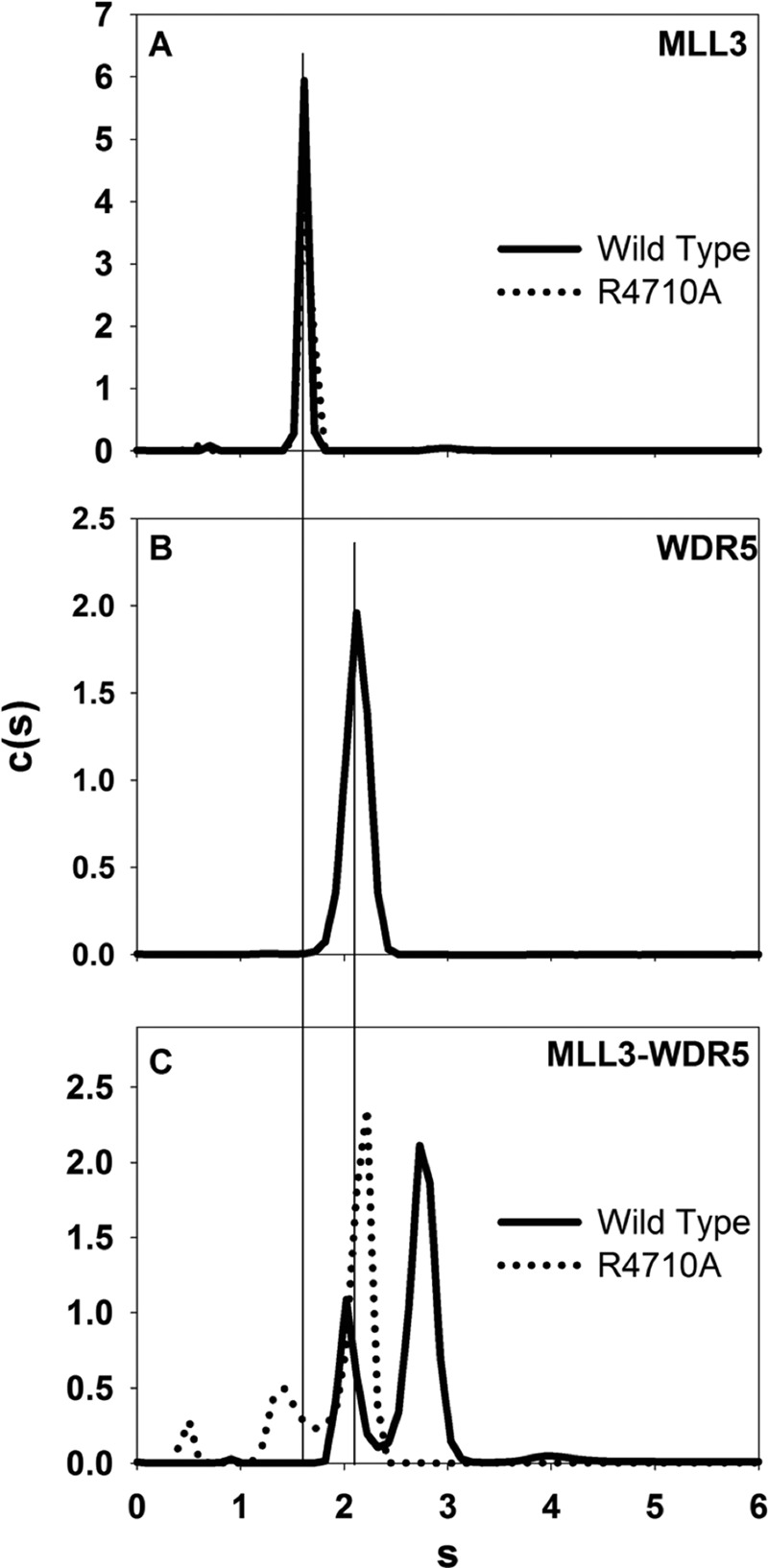
**Win motif is required for the interaction of MLL3 and WDR5.** *A,* comparison of the *c*(*s*) distributions for the isolated MLL3^WT^ (*solid black line*) and MLL3^R4710A^ variant (*dashed line*) SET domains run at the same concentration. *B, c*(*s*) distribution of full-length wild type WDR5. *C,* comparison of *c*(*s*) distributions for the interaction of the wild type (*solid black line*) or the R4710A variant (*dashed line*) MLL3 SET domain with WDR5.

To further test the hypothesis that MLL3 interacts with RAD in the absence of WDR5, we compared the hydrodynamic properties of the MLL3-WRAD and MLL3-RAD complexes using SV-AUC. When MLL3 was mixed with WRAD, the complex sedimented with an *s* value of 5.2 (Fig. 3*C* and Table 2), which is similar to the *s* value previously reported for the MLL1 core complex (5.3 s) (20, 22). The MLL3-WRAD sedimentation coefficient did not change over a 5-fold dilution range, suggesting the complex is stable over the time course of sedimentation (Fig. 3*C*). When MLL3 was mixed with RAD, the predominant species that was observed sedimented at 4.5 s (Fig. 3*D*), which is greater than the *s* value for the RAD subcomplex at 3.9 s (Fig. 3*E* and Table 2) or the MLL3 SET domain at 1.6 s (Fig. 4*A*, *solid black line,* and Table 2). Dilution over a 5-fold concentration range did not alter the *s* value of this peak, suggesting that the 4.5-s species is the MLL3-RAD complex and is stable on the time scale of sedimentation. Taken together, these results demonstrate that MLL3 forms complexes with both WRAD and RAD subcomplexes with similar stabilities.

**TABLE 2:** **Summary of sedimentation velocity data**

Complex	*s**^a^*	*s*_20,_*_w_**^b^*	*f*/*f*_0_*^c^*
MLL3^WT^	1.6 ± 0.01	2.3	1.4
MLL3^WT^-W	2.7 ± 0.02	3.8	1.5
RAD	3.9 ± 0.01	5.5	1.8
MLL3^WT^-RAD	4.5 ± 0.04	6.3	1.8
MLL3^WT^-WRAD	5.2 ± 0.03	7.3	1.8

*^a^* Experimental sedimentation coefficients were determined at 10 °C ± S.D. from duplicate or triplicate experiments at the same concentration.

*^b^* Standard sedimentation coefficient (*s*_20,_*_w_*) after correcting for water at 20 °C in the program SEDNTERP is shown.

*^c^* Frictional ratios were calculated using the program SEDNTERP as described under “Experimental Procedures.”

##### Win Motif Is Required for Interaction of WDR5 with MLL3

We and others previously identified the highly conserved arginine containing Win motif as the region responsible for the interaction of MLL1 with WDR5 (21, 26, 43). Mutation of the conserved arginine in the MLL1 Win motif disrupts a key interaction network with WDR5 resulting in core complex disassembly and loss of H3K4 di-methylation activity (23, 26). The Win motif is conserved in MLL3, and previous experiments revealed that a peptide derived from the MLL3 Win motif binds to WDR5 with greater affinity than that of any other human SET1 family Win motif peptide (23). To determine whether the MLL3 Win motif is necessary for the interaction of WDR5 with the MLL3 core complex, we used SV-AUC to compare complex formation between wild type MLL3 and an MLL3 construct in which the crucial arginine (Arg-4710) of the MLL3 Win motif was replaced by alanine. SV-AUC of the isolated wild type and R4710A MLL3 SET domains showed that they sedimented with identical *s* values at 1.6 s, indicating that the mutation did not alter the gross hydrodynamic shape of the protein (Fig. 4*A*). We next compared binary complex formation of wild type and variant MLL3 SET domains with WDR5. SV-AUC of isolated WDR5 showed an *s* value of 2.2 s (Fig. 4*B*). When wild type MLL3 was mixed with WDR5, two peaks are observed in the *c*(*s*) distribution at 2.2 and 2.7 s (Fig. 4*C, solid black line*). The peak at 2.2 s corresponds to free WDR5, and the peak at 2.7 s corresponds to a 1:1 complex between WDR5 and MLL3, which did not shift when diluted 3.5-fold (data not shown). These results suggest that MLL3 forms a 1:1 complex with WDR5 that is stable on the time scale of sedimentation. In contrast, when the R4710A variant of MLL3 was mixed with WDR5, the species corresponding to the complex at 2.7 s was absent, and the profile instead showed peaks corresponding to free MLL3 and WDR5 at 1.5 and 2.2 s, respectively (Fig. 4*C, dashed line*). Taken together, these results suggest that the conserved Arg-4710 of the MLL3 Win motif is required for the interaction of MLL3 with WDR5.

##### Win Motif Is Required for the Stable Interaction of WDR5 with the MLL3 Core Complex

**FIGURE 5.**
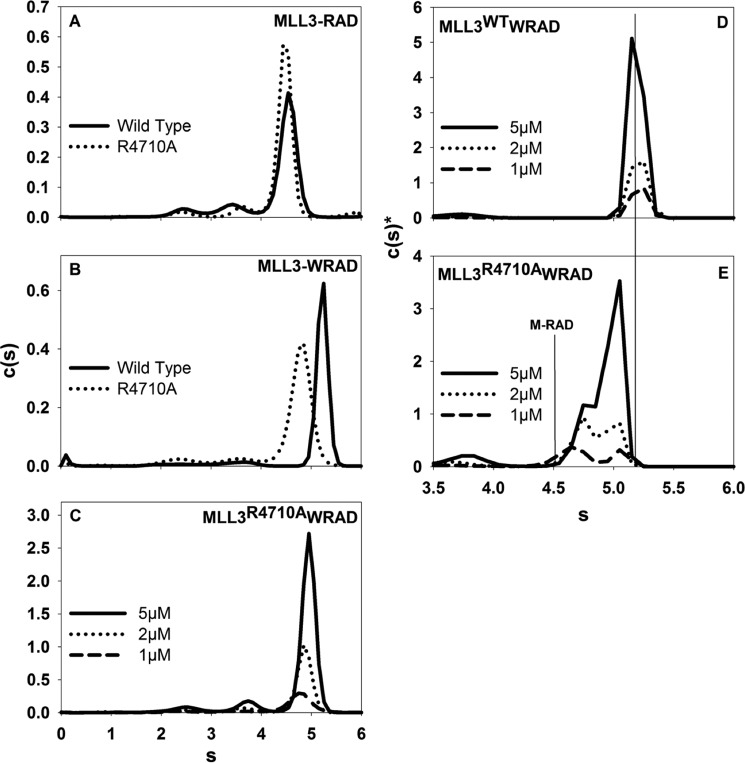
**Win motif contributes to the stabilization of WDR5 within the core complex.** *A* and *B*, comparison of *c*(*s*) distributions of the wild type (*solid black line*) or the R4710A variant (*dashed line*) MLL3 SET domain assembled with the RAD (*A*) or WRAD (*B*) subcomplex. *C,* comparison of the *c*(*s*) distributions of the R4710A variant MLL3 SET domain assembled with WRAD at different concentrations. *D* and *E*, comparison of weighted *c*(*s*) distributions (*c*(*s*)*) (Bayesian analysis) of the wild type (*D*) or R4710A variant (*E*) MLL3 SET domain assembled with WRAD.

We previously found that the MLL1 Win motif was necessary for interaction with WDR5 and assembly of the holo-MLL1 core complex (21). To determine the role of the Win motif in MLL3 core complex assembly, we compared wild type and R4710A MLL3 variants for their ability to assemble with RAD and WRAD using SV-AUC. When wild type MLL3 was mixed with RAD, a stable complex was formed that sediments at 4.5 s (Figs. 3*D* and 5*A, solid line*). A peak with the same sedimentation value was observed when the R4710A MLL3 variant was mixed with RAD (Fig. 5*A*, *dotted line*), indicating that Arg-4710 of the MLL3 Win motif is not required for MLL3-RAD complex formation.

In contrast, whereas the wild type MLL3 interacts with WRAD with an *s* value of 5.2 s that is independent of complex concentration (Figs. 3*C* and 5*B, solid black line*), mixture of the R4710A MLL3 variant with WRAD results in a complex with a peak that is shifted to 4.9 s that is modestly concentration-dependent (Fig. 5, *B, dashed line,* and *C*). Given that the RAD complex interacts in a stable manner with MLL3^R4710A^ variant, these results suggest that the peak at 4.9 s likely represents an equilibrium mixture between the MLL3^R4710A^-WRAD complex and MLL3^R4710A^-RAD complex, which cannot be resolved within the signal-to-noise of the data. To further test this hypothesis, we re-analyzed the SV-AUC data for wild type and R4710A MLL3-WRAD complexes using a Bayesian approach, which weight the data in the *c*(*s*) analysis based on the known sedimentation coefficient of the wild type MLL3-WRAD complex at 5.2 s (44). When the Bayesian approach was used to analyze the SV-AUC data from wild type MLL3-WRAD complex, a single peak was observed at 5.2 s at each of the three concentrations tested (Fig. 5*D*). In contrast, when the same Bayesian approach was used to analyze the MLL3^R4710A^-WRAD complex, the 4.9-s species resolves into two peaks with concentration-dependent sedimentation behavior suggesting that the peak represents an equilibrium mixture of MLL3-RAD and MLL3-WRAD complexes (Fig. 5*E*). These results are consistent with the hypothesis that mutation of the MLL3 Win motif alters the equilibrium of WDR5 binding to the MLL3 core complex.

Taken together, these results suggest that the Win motif is required for stable interaction of WDR5 to the MLL3 core complex. However, unlike that observed with the MLL1 core complex, WDR5 is not required to bridge the interaction between MLL3 and the RAD subcomplex.

##### MLL3 Win Motif Is Required for the Inhibition of the MLL3 Core Complex by WDR5

**FIGURE 6.**
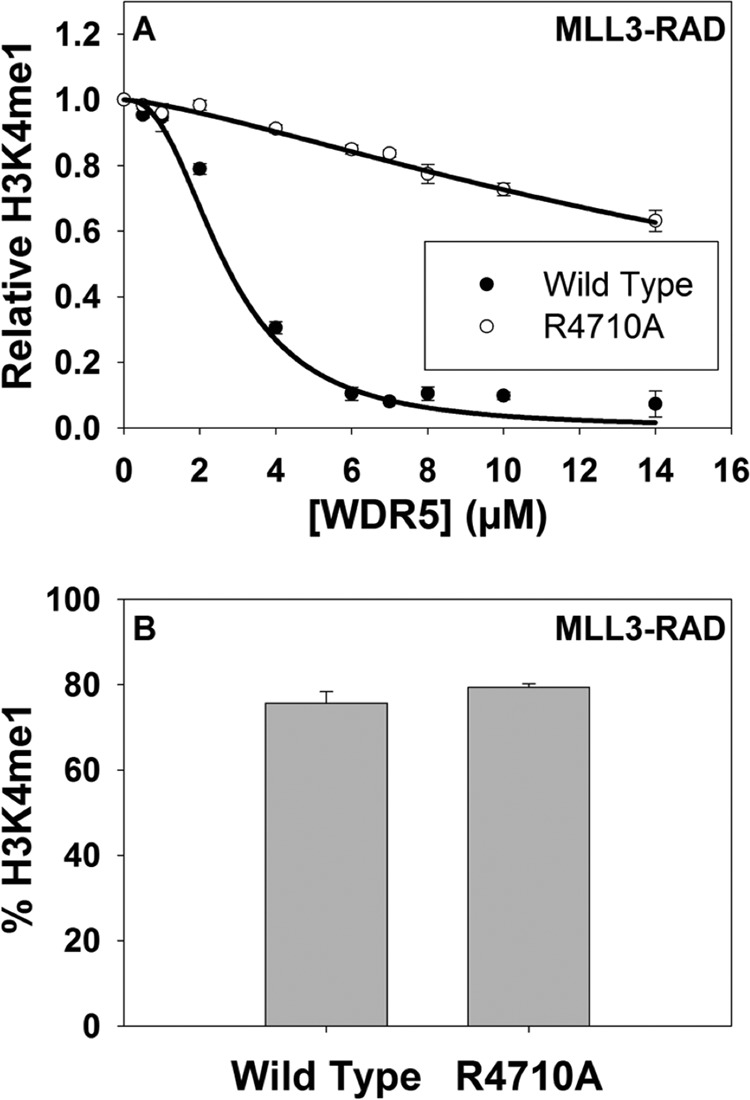
**WDR5 partially inhibits the activity of the MLL3 core complex.** *A*, activity assays of the MLL3^WT^-RAD (*filled circles*) or MLL3^R4710A^-RAD (*open circles*) complexes with increasing amounts of WDR5. Each point represents the mean percentage of total integrated area for each sample in MALDI-TOF reactions after 30 min. *Error bars* represent ± S.E. from triplicate measurements. The data are normalized to the amount of H3K4me1 activity that either the MLL3^WT^-RAD or the MLL3^R4710A^-RAD complexes catalyzed at 30 min when no WDR5 was present. Inhibition curves are fit as described under the “Experimental Procedures.” *B, graph* indicates that the MLL3-RAD complexes assembled with the wild type or the R4710A variant catalyze the same amount of H3K4 monomethylation (H3K4me1) after a 30-min reaction as determined by MALDI-TOF assays. *Error bars* represent ± S.E. from triplicate experiments.

The results of the activity assays demonstrate that the MLL3 core complex assembled without WDR5 is more active than the complex assembled with WDR5. This observation suggests that WDR5 partially inhibits the activity of the MLL3 core complex, consistent with previous results (24). To further test this hypothesis, we assembled the MLL3-RAD complex with increasing amounts of WDR5 and assayed methyltransferase activity using quantitative MALDI-TOF mass spectrometry. We found that WDR5 inhibited the activity of the MLL3-RAD complex in a concentration-dependent manner with an IC_50_ value of 2.7 ± 0.3 μm (Fig. 6*A*, *filled circles*). In contrast, when WDR5 was titrated into the MLL3-RAD complex assembled with the R4710A Win motif variant, only modest inhibition was observed (Fig. 6*A*, *open circles*). These differences cannot be attributed to differences in the overall activity of MLL3-RAD complexes assembled with the wild type or R4710A MLL3 variants, as assays show they have similar overall levels of activity (Fig. 6*B*). These results demonstrate that the Arg-4710 of the MLL3 Win motif is required for the inhibitory effect of WDR5 on the MLL3 core complex.

##### WDR5 Inhibits the Activity of WRAD within the MLL3 Core Complex

We previously found that WRAD, in the absence of the MLL1 SET domain, possesses a cryptic H3K4 methyltransferase activity (20, 45). We also found that when WRAD is in complex with a catalytically inactive MLL1 SET domain, weak H3K4 dimethylation could be observed, suggesting that the MLL1 core complex uses two distinct active sites to catalyze multiple H3K4 methylation (20). The discovery that the MLL3 core complex catalyzes only H3K4 monomethylation, however, has raised questions about the enzymatic role of WRAD within the MLL3 core complex. Either the MLL3 SET domain is solely responsible for the observed H3K4 monomethylation activity of the MLL3 core complex (one active site model) or WRAD and the MLL3 SET domain each catalyze H3K4 monomethylation within the complex (two active site model) (46). To distinguish these hypotheses, we compared AdoMet binding and enzymatic activities of MLL3-WRAD and MLL3-RAD complexes assembled with wild type or the N4848A MLL3 variant using radiometric assays. Asn-4848 is part of the highly conserved NHS motif found in all SET domain proteins, and its replacement with alanine has been shown to abolish AdoMet binding in the SET domain of all human SET1 family members (24).

**FIGURE 7.**
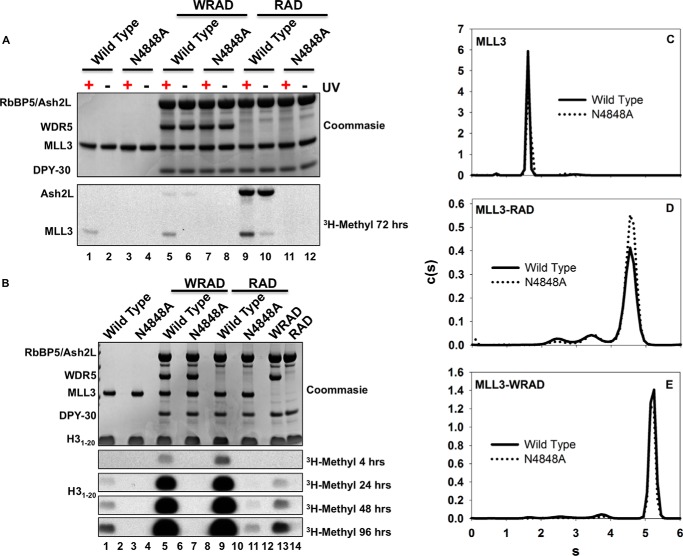
**MLL3 core complex utilizes two active sites.** *A,* UV light was used to cross-link [^3^H]AdoMet to wild type or N4848A variant MLL3 SET domains in isolation or assembled with WRAD/RAD. The *upper panel* shows a Coomassie Blue-stained SDS-polyacrylamide gel, and the *lower panel* shows the [^3^H]methyl incorporation after a 72-h exposure to film by fluorography. *B,* comparison of methyltransferase activities among the wild type or the N4848A variant MLL3 SET domain alone or when assembled with WRAD/RAD. The *upper panel* shows a Coomassie Blue-stained SDS-polyacrylamide gel, and the *lower panels* show [^3^H]methyl incorporation after 4, 24, 48, or 96 h by fluorography. *C–E*, the *c*(*s*) analysis compares the wild type (*solid black lines*) or the N4848A variant (*dotted lines*) of the isolated MLL3 SET domain (*C*), the MLL3-RAD complex (*D*), or the MLL3-WRAD complex (*E*).

To confirm that the N4848A mutation disrupts AdoMet binding to the SET domain within the MLL3 core complex, we used UV cross-linking to compare the [^3^H]AdoMet binding to the wild type and mutant MLL3 in the presence and absence of WRAD/RAD. We found that although the isolated wild type MLL3 SET domain showed UV-dependent cross-linking of [^3^H]AdoMet (Fig. 7*A*, *lane 1*), the N4848A variant showed no cross-linking (Fig. 7*A*, *lane 3*). Similarly, in the presence of WRAD, the wild type MLL3 SET domain showed UV-dependent cross-linking of [^3^H]AdoMet (Fig. 7*A*, *lane 5*) but the N4848A variant did not (*lane 7*), suggesting that WRAD does not rescue the AdoMet-binding defect of the N4848A substitution (Fig. 7*A*, compare *lanes 5–7*). Interestingly, in the absence of WDR5, the MLL3-RAD complex showed greater UV-dependent cross-linking of [^3^H]AdoMet (Fig. 7*A*, *lane 9*) compared with that of the MLL3-WRAD complex (*lane 5*). The MLL3-RAD complex also showed greater Ash2L auto-methylation, which is UV-independent (Fig. 7*A*, *upper bands, lanes 9* and *10* compared with *lanes 5* and *6*). These results suggest that AdoMet affinity for the MLL3 SET domain is increased when WDR5 is omitted from the complex. Despite this increased binding, however, replacement of Asn-4848 with alanine in the MLL3 SET domain abolishes all AdoMet cross-linking (Fig. 7*A*, *lane 11*). These results suggest that WRAD/RAD does not rescue the AdoMet-binding defect of the N4848A MLL3 SET domain.

We next compared enzymatic activities of the wild type and N4848A MLL3 SET domains in the presence and absence of WRAD/RAD. When the isolated wild type MLL3 SET domain was incubated with a histone H3 peptide and [^3^H]AdoMet, relatively weak H3K4 methylation activity was observed (Fig. 7*B*, *lane 1*). In contrast, when the isolated N4848A MLL3 SET domain was assayed, no activity could be observed, even after a 96-h exposure to film (Fig. 7*B*, compare *lanes 1–3*). Comparison of sedimentation coefficient distributions by SV-AUC showed that the wild type and N4848A MLL3 SET domains have similar hydrodynamic properties, suggesting that the mutation does not disrupt the overall fold of the protein (Fig. 7*C*).

We then compared complex formation and enzymatic activities of MLL3 core complexes assembled with wild type or the N4848A MLL3 variant in the presence and absence of WDR5. Comparison of sedimentation coefficient distributions of MLL3-WRAD and MLL3-RAD complexes assembled with wild type or the N4848A variant revealed that they were essentially identical, suggesting that replacement of Asn-4848 with alanine does not disrupt complex assembly (Fig. 7, *D* and *E*). In enzymatic assays, although we observed relatively robust H3K4 methylation when wild type MLL3 was assembled with WRAD (Fig. 7*B*, *lane 5*), the complex assembled with the N4848A variant was inactive, even after a 96-h exposure to film (Fig. 7*B*, *lane 7*). This result suggests that the activity observed by the MLL3-WRAD complex is dependent on the enzymatic activity of the MLL3 SET domain, which is consistent with the one active site model. However, the same concentration of WRAD in the absence of the MLL3 SET domain displayed as much or more activity than the isolated wild type MLL3 SET domain (compare *lanes 1* and *13*), suggesting that WRAD is enzymatically active under the assayed conditions and that the N4848A MLL3 SET domain inhibits the activity of WRAD within the complex (compare *lanes 7* and *13*). In support of this conclusion, we found that inhibition of WRAD by the N4848A MLL3 SET domain could be partially overcome by omitting the WDR5 subunit from the complex. When the N4848A MLL3 variant was incubated with the RAD complex, weak activity was observed (Fig. 7*B*, *lane 11*). The amount of activity was considerably less than that of the MLL3-RAD complex assembled with wild type MLL3 (Fig. 7*B*, *lane 9*) but was greater than that observed with the MLL3^N4848A^-WRAD complex (*lane 7*). These results suggest that the majority of H3K4 monomethylation activity of the MLL3 core complex is due to the activity of the MLL3 SET domain and that WDR5 inhibits the activity of WRAD within the MLL3 core complex.

##### Solution Structures of the MLL3 Core Complex by SAXS

**FIGURE 8.**
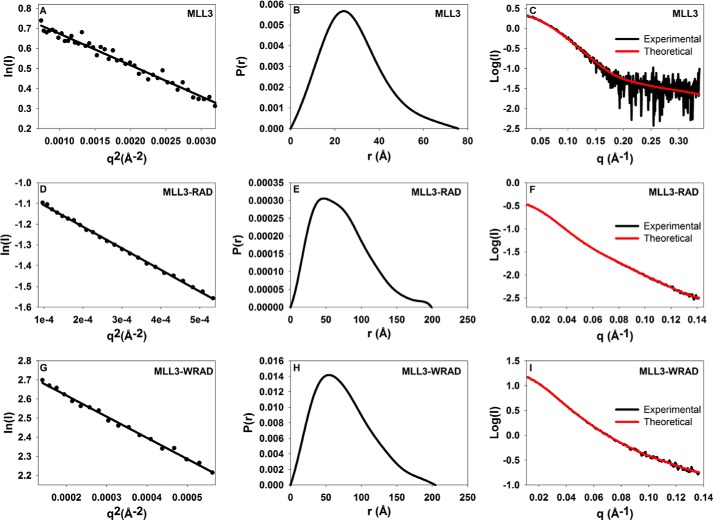
**Biological SAXS characterization of MLL3 core complexes.** *A*, *D*, and *G*, Guinier plots of the data showing a linear fit that satisfies *qR_g_* of ≤1.3, where *R_g_* is the radius of gyration, and *q* (momentum transfer) = 4πsin(θ)/λ, where 2θ is the scattering angle, and λ is the x-ray wavelength. The data are plotted as the natural log of the scattering intensity (*I*) as a function of the square of momentum transfer (*q*). *B, E, H,* Plots of the pair distance distribution function, *P*(*r*), for MLL3 (*B*), MLL3-RAD (*E*), and MLL3-WRAD (*H*). The maximum intraparticle distance (*D*_max_) for each plot is reported in Table 3. *C, F,* and *I*, plots of the fit of the simulated scattering from the calculated envelopes overlaid with the experimental scattering data. The data are plotted as the log of the intensity (*I*) as a function of *q*. The *q* range used for structural determination is determined such that *q*_max_ ≤*R_g_*/8.

Structural information about the MLL3 core complex is limited as no high resolution structures of MLL3 or MLL3 complexes are currently available. In addition, although cryo-EM reconstructions of the budding yeast SET1 complex and human MLL1 core complex have been reported (47), there is currently no information about solution structures and dynamics of any SET1 family complex. To begin to gain an understanding of the solution structural properties of MLL3 core complexes, SAXS measurements were made of free MLL3 and MLL3 within the context of WRAD/RAD. SAXS scattering data for free MLL3, MLL3-WRAD, and MLL3-RAD complexes were plotted as Guinier curves, all of which displayed linearity at low *q* (Fig. 8, *A*, *D,* and *G*), consistent with a soluble non-aggregated sample (33). For each sample, minimal concentration dependence was observed in *R_g_*, indicating that the oligomerization state of each sample did not change over the concentration range used. Using the Porod volume method (48), molecular weights determined from scattering data gave values of 27, 212, and 223 kDa for MLL3, MLL3-RAD, and MLL3-WRAD complexes, respectively, which were in reasonable agreement with actual values based on their protein sequences for the MLL3 monomer (26 kDa), a 1:1:1:2 stoichiometric complex for MLL3-RAD (176 kDa), and a 1:1:1:1:2 stoichiometric complex for MLL3-WRAD (207 kDa). These results suggest that MLL3 and the MLL3-WRAD/RAD complexes are monodisperse under these assay conditions.

**FIGURE 9.**
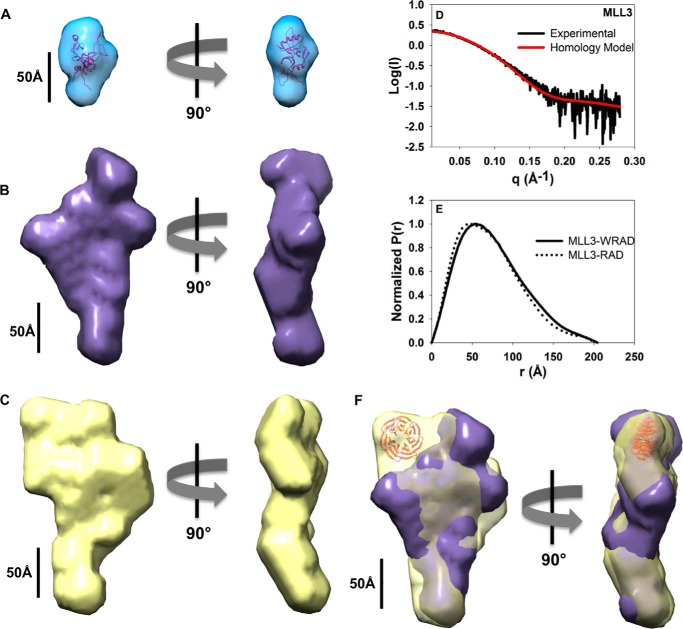
**Solution structures of MLL3 core complexes.** The *ab initio* solution structures of MLL3(4690–4911) (*A*), the MLL3-RAD complex (*B*), and the MLL3-WRAD complex (*C*) were contoured at 20 Å. The final models represent the refined average model from 9 to 10 individual models generated using programs from the ATSAS package (56) as described under “Experimental Procedures.” *A*, MLL3 homology model was generated using Modeler (40) and was fit into the MLL3 envelope. *D,* predicted solution scattering profile of the MLL3 homology model from the FoxS server overlaid with the experimental MLL3 scattering data. *E,* overlay of the normalized *P*(*r*) distributions from the MLL3-WRAD (*solid line*) and MLL3-RAD (*dotted line*) complexes. *F*, rigid body alignment of the MLL3-RAD and MLL3-WRAD envelopes. The WDR5 crystal structure (PDB code 2H14 (49)) is fit into a region of the MLL3-WRAD structure that is missing from the MLL3-RAD structure in the alignment.

The overall radius of gyration, *R_g_*, determined from the slope of the Guinier curve was 22.4 Å for free MLL3, 55.7 Å for the MLL3-RAD complex, and 57.4 Å for the MLL3-WRAD complex (Table 3). These data suggest that the MLL3-WRAD and MLL3-RAD complexes have relatively similar dimensions in solution. Indeed, comparison of interatomic distribution functions (*P*(*r*)) of the MLL3-WRAD and MLL3-RAD complexes reveals similar maximum inter-particle distances (*D*_max_) suggesting that the particles are extended and have similar overall dimensions in solution (Fig. 8, *E* and *H*). Consistent with this hypothesis, the MLL3-RAD and MLL3-WRAD complexes had identical elution volumes during size exclusion chromatography and identical *f*/*f*_0_ values from SV-AUC analysis, suggesting each has a similar Stokes radius. Despite these similarities, there were modest deviations in the *P*(*r*) distributions of the MLL3-RAD and MLL3-WRAD complexes, likely representing the contribution of WDR5 to the scattering (Fig. 9*E*).

**TABLE 3:** **Summary of biological SAXS data processing and ab initio model building**

Sample	*R_g_* Guinier*^a^*	*R_g_ P*(*r*)*^b^*	*D*_max_*^c^*	GNOM total estimate*^d^*	NSD*^e^*	*S*_predicted_*^f^* (% discrepancy)	Calculated molecular mass*^g^* (expected) (kDa)
	Å	Å	Å				
MLL3	22.4 ± 0.66	22.1 ± 0.54	75.7	0.77	0.52 ± 0.01	1.6 (1.9)	27 (26)
MLL3-RAD	55.7 ± 0.16	57.1 ± 0.10	198.9	0.80	0.68 ± 0.03	4.8 (6.2)	212 (176)
MLL3-WRAD	57.4 ± 1.80	59.7 ± 0.94	204.3	0.83	0.64 ± 0.02	5.5 (5.1)	223 (207)

*^a^* Radius of gyration (*R_g_*) values were determined with a linear fit to the scattering intensity at low *q* that satisfies the relationship *qR_g_* <1.3.

*^b^* Radius of gyration reported from the *P*(*r*) distribution.

*^c^ D*_max_ was chosen where *P*(*r*) approached zero.

*^d^* GNOM total estimates were all rated as “good.”

*^e^* The NSD ± the variance was reported by DAMAVER. NSD values of <0.7 indicate good stability of the solution.

*^f^* HydroPro was used to calculate the theoretical sedimentation coefficient of each *ab initio* model as described under “Experimental Procedures.” The percent discrepancy compared with the actual sedimentation coefficient is indicated in parentheses.

*^g^* The Porod volume method was used to calculate the molecular weight from the scattering data. The expected molecular weight based on amino acid sequence is indicated in parentheses.

Three-dimensional *ab initio* molecular envelopes were reconstructed using DAMMIF (35) without symmetry restraints from SAXS data collected for the MLL3 SET domain, MLL3-WRAD and MLL3-RAD complexes, and each resulted in highly reproducible models. The 10 randomly generated models for the MLL3 SET domain superimposed with a NSD value of 0.52 ± 0.01 (Table 3), which is indicative of a stable solution (36). Likewise, the NSD values for superimposed models for the MLL3-RAD and MLL3-WRAD complexes were 0.68 ± 0.03 and 0.64 ± 0.02, respectively (Table 3). Calculated scattering profiles from the refined average *ab initio* models fit the experimental scattering data well (Fig. 8, *C, F,* and *I*). Validation of the molecular envelopes can be accomplished by comparison of the experimental scattering data to that of theoretical scattering profiles calculated from available crystal structures. Although the crystal structure of the MLL3 SET domain has not been reported, the structure of the homologous MLL1 SET domain has been reported at 2.0 Å (39). We generated a homology model of the MLL3 SET domain and found that it fit well within the refined average *ab initio* molecular envelope derived from scattering data of the MLL3 SET domain (Fig. 9*A*). We also found good agreement between its theoretical scattering profile calculated from the homology model and the experimental SAXS data (Fig. 9*D*). Because crystal structures of MLL3 complexes are currently lacking, we compared theoretical sedimentation coefficients calculated from the average *ab initio* molecular envelopes using HydroPro (38) to that of experimentally determined sedimentation coefficients derived from SV-AUC. The results show that in each case there is generally good agreement between the theoretical and experimental sedimentation coefficients (Table 3), suggesting that the *ab initio* molecular envelopes are accurate low resolution representations of the average structures in solution.

The molecular envelopes for the MLL3-WRAD and MLL3-RAD complexes are asymmetric and have elongated and flattened clavate shapes with similar dimensions (Fig. 9, *B* and *C*). The general appearance agrees well with previous cryo-EM observations of the MLL1 core complex, which has been described to have an elongated Y-shaped structure (47). These shapes are also consistent with the relatively high frictional coefficients observed in SV-AUC experiments (Table 2). Rigid body superposition of the two models show generally good agreement, with the exception of an additional lobe of density that is unaccounted for in the MLL3-WRAD envelope (Fig. 9*F*). We suggest that this density represents the location of WDR5 within the complex. Indeed docking of the WDR5 crystal structure (PDB code 2H14 (49)) in this region accommodated the density well (Fig. 9*F*). Taken together, these results show that the MLL3-core complex forms in the presence and absence of WDR5 and that WDR5 does not significantly alter the overall conformation of the MLL3 core complex.

## Discussion

The members of the WRAD subcomplex act as regulators of H3K4 methylation by interacting with and altering the activity of SET1 family members and by enzymatically contributing to total H3K4 methylation activity (20, 24). However, the specific contributions of the individual WRAD subunits are not completely understood. This is further complicated by the observation that different SET1 family members share requirement for some WRAD subunits but differ in their requirement for others *in vitro*, particularly WDR5 (24). In this investigation, we set out to further characterize the role of WDR5 in the MLL3 core complex. Unlike that for MLL1, we find that removal of WDR5 from the MLL3 core complex results in a significant stimulation in H3K4 methyltransferase activity and that MLL3 can interact with the RAD subcomplex in the absence of the Win motif-WDR5 interaction. These results suggest that WDR5 acts as an inhibitor of the H3K4 methyltransferase activity of the MLL3 core complex. Although the mechanism by which WDR5 inhibits MLL3 core complex activity remains unknown, the core complexes assembled with and without WDR5 have similar overall structures in solution as determined by SV-AUC and biological SAXS, suggesting large structural rearrangements of the complex do not account for the differences. However, because we did observe increased AdoMet cross-linking in the absence of WDR5, it is possible that the increased activity could be due in part to increased AdoMet binding to the MLL3 SET domain. Why this effect is specific to the MLL3 SET domain and not the other human SET1 family members is unknown.

The observation that RbBP5 and Ash2L interacts with the MLL3 SET domain in the absence of WDR5 is surprising given the following: 1) the requirement of WDR5 for the interaction between MLL1 and the RbBP5/Ash2L heterodimer (20, 21); 2) the conservation of the MLL3 Win motif; and 3) our previous demonstration that WDR5 interacts with an MLL3 Win motif peptide with 51-fold greater affinity than that of a comparable MLL1 Win motif peptide (23). These results suggest that amino acid sequences unique to the MLL3 SET domain have evolved to stabilize the interaction with RbBP5/Ash2L in the absence of WDR5. Analysis of Kabuki syndrome missense mutations when introduced into the MLL1 SET domain resulted in the identification of a surface, called the Kabuki interaction surface, that is required for interaction of MLL1 with the RbBP5/Ash2L heterodimer within the MLL1 core complex (22). However, Kabuki interaction surface residues are conserved among SET1 family members from yeast to humans and therefore cannot account for the unique ability of MLL3 to interact with the RA heterodimer in the absence of WDR5. Another study identified a SET domain basic patch that was sufficient for the interaction of the human SETd1A SET domain with RbBP5 in the absence of WDR5 (50). Although the basic patch is conserved among SETd1A/B orthologs, it is not conserved in MLL 1–4 paralogs (50), suggesting that this region also cannot explain why MLL3 interacts with RbBP5/Ash2L independently of WDR5.

Despite the differences in affinity for the RbBP5/Ash2L heterodimer, both MLL1 and MLL3 require the Win motif for stable interaction of WDR5 within the core complex (this work and Ref. 21). This suggests that WDR5 serves some common critical function in both core complexes. Indeed, WDR5 has been shown to bind long non-coding RNAs, such as HOTTIP (51, 52), and PIWI-interacting RNAs, such as GAS5 (53), and to recruit SET1 family members to subsets of target genes. Therefore, the MLL3 Win motif-WDR5 interaction may have been retained within the MLL3 core complex for gene targeting.

These findings raise a number of interesting questions. Does WDR5 regulate the activity of the MLL3 core complex *in vivo*? At what genes does WDR5 co-localize with MLL3 and do these genes show differentially reduced levels of H3K4 methylation compared with genes where they do not co-localize? A recent report suggests that the activity of the MLL3 core complex could be regulated by reversible phosphorylation of the RbBP5 subunit at Ser-350 (54). This raises the possibility that RbBP5 phosphorylation results in eviction of the WDR5 subunit of the MLL3 core complex, increasing its activity. However, assembly of the MLL3 core complex with the phospho-mimic S350E RbBP5 variant does not alter enzymatic activity or complex integrity,^3^ suggesting either that the glutamate does not sufficiently mimic phosphoserine or that some other mechanism is involved. It is also possible that MLL3 core complex assembly is spatially regulated. For example, the progestin and AdipoQ receptor member 3 (PAQR3) was found to sequester WRAD components at the Golgi membrane through direct binding to WDR5, RbBP5, and Ash2L resulting in reduced H3K4 methylation (55). Additionally, PAQR3 can specifically interfere with the interaction between WDR5 and MLL1 and reduce H3K4 methylation *in vitro* (55). However, it is not currently known whether there are mechanisms that specifically regulate WDR5 binding to the MLL3 core complex.

In summary, we have established that the MLL3 core complex assembles independently of WDR5 and that WDR5 partially inhibits the activity of MLL3 by interaction with the complex. This information will facilitate future studies to identify the cellular machinery involved in regulating MLL3 core complex assembly and the molecular mechanisms by which WDR5 reduces MLL3 methyltransferase activity. In addition, this work adds to our understanding of how additional regulatory mechanisms can influence the methyltransferase activity of SET1 family members.

## Author Contributions

S. A. S. performed all the experiments, processed and interpreted the data, and generated the figures as well as drafted the manuscript. M. S. C. supervised the project and drafted the manuscript.

## Footnotes

3 S. A. Shinsky, M. Jaskolka, and M. S. Cosgrove, unpublished data.

## Footnotes

2 The abbreviations used are:

SET: suppressor of variegation, enhancer of zeste, trithorax
AdoMet: *S*-adenosyl-l-methionine
BisTris: 2-[bis(2-hydroxyethyl)amino]-2-(hydroxymethyl)propane-1,3-diol
SAXS: small angle x-ray scattering
PDB: Protein Data Bank
NSD: normalized spatial discrepancy
SV: sedimentation velocity
AUC: analytical ultracentrifugation.
